# Splenorenal Manifestations of *Bartonella henselae* Infection in a Pediatric Patient

**DOI:** 10.1155/2016/7803832

**Published:** 2016-04-05

**Authors:** Taylor Rising, Nicholas Fulton, Pauravi Vasavada

**Affiliations:** ^1^Northeast Ohio Medical University, 4209 State Route 44, Rootstown, OH 44272, USA; ^2^University Hospitals Case Medical Center, 11100 Euclid Avenue, Cleveland, OH 44106, USA

## Abstract

*Bartonella henselae* is a bacterium which can cause a wide range of clinical manifestations, ranging from fever of unknown origin to a potentially fatal endocarditis. We report a case of* Bartonella henselae* infection in a pediatric-aged patient following a scratch from a kitten. The patient initially presented with a prolonged fever of unknown origin which was unresponsive to antibiotic treatment. The patient was hospitalized with worsening fevers and night sweat. Subsequent ultrasound imaging demonstrated multiple hypoechoic foci within the spleen. A contrast-enhanced CT of the abdomen and pelvis was also obtained which showed hypoattenuating lesions in the spleen and bilateral kidneys.* Bartonella henselae* IgG and IgM titers were positive, consistent with an acute* Bartonella henselae* infection. The patient was discharged with a course of oral rifampin and trimethoprim-sulfamethoxazole, and all symptoms had resolved following two weeks of therapy.

## 1. Introduction

*Bartonella henselae* commonly presents as fever and localized lymphadenopathy in children or adolescents with a history of exposure to a scratch from a kitten or cat. The most commonly involved lymph nodes are axillary and epitrochlear, presumably because the majority of contact with cats occurs with the hands [1]. The bacterium is an aerobic Gram-negative bacillus which is thought to disseminate hematogenously, most commonly to the liver and spleen [1, 2]. Hepatosplenic involvement may manifest clinically as periumbilical or upper abdominal pain, weight loss, and hepatosplenomegaly [1]. Typical imaging findings include hypoechoic lesions in the liver and spleen by ultrasound and hypoattenuating lesions on CT [3]. More recently, renal microabscesses as a result of* Bartonella henselae* infection have also been reported [4]. We report a patient with splenorenal involvement of* Bartonella henselae* on ultrasound and CT in a pediatric patient.

## 2. Case Presentation

A previously healthy six-year-old female presented with complaint of fevers up to 101 degrees Fahrenheit, as well as dry cough, intermittent periumbilical abdominal pain, and night sweat. She was taken to her primary care physician, who diagnosed the patient with a urinary tract infection and she was sent home with a 5-day course of oral amoxicillin. The patient returned to the emergency room 2 days after completion of the antibiotics with persistent fevers and cough. Urinalysis and complete blood count were unremarkable. A chest radiograph showed perihilar peribronchial thickening without focal consolidation. The patient was discharged home with a presumed diagnosis of atypical pneumonia and given a 5-day course of oral azithromycin. Following completion of the azithromycin, the patient had improvement in her cough but still had persistent fevers, prompting another visit to the emergency department the day after completing her antibiotic course. A repeat chest radiograph, complete blood count, urinalysis, and renal function panel were normal. C-reactive protein was elevated at 6.65 mg/dL (normal < 0.80) and erythrocyte sedimentation rate was elevated at 70 mm/h (normal 0–13). The patient was hospitalized for further evaluation of her fever.

Blood and urine cultures on the date of admission did not demonstrate bacterial growth. Further investigation into the patient's history did not demonstrate any sick contacts but did have exposure to a family member who had been recently incarcerated. The patient did also have recent contacts with new dogs and kittens prior to the onset of fever, and the patient did suffer a scratch to the chest from one of the kittens which did not require medical care.

**Figure 1:**
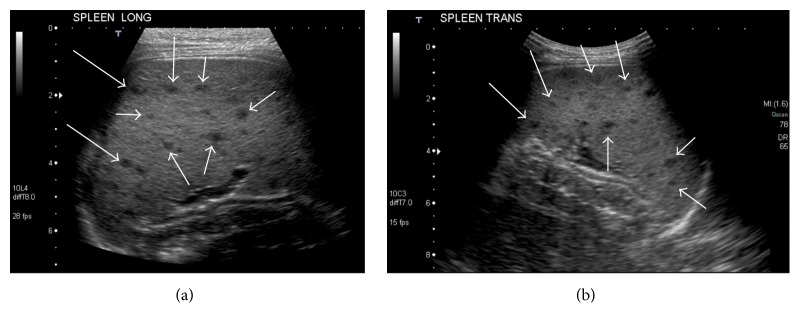
Longitudinal and transverse ultrasound images of the left upper quadrant demonstrate the spleen to be enlarged. Multiple small hypoechoic lesions (arrows) are seen within the spleen.

**Figure 2:**
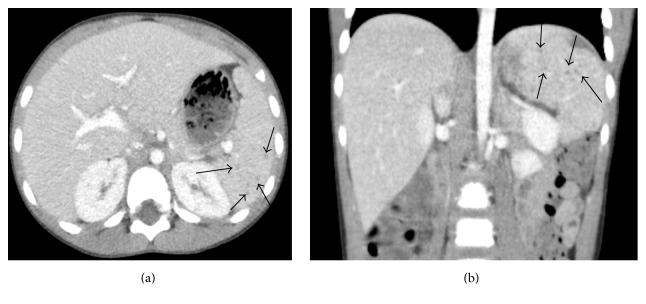
Axial and coronal contrast-enhanced CT images through the upper abdomen demonstrate an enlarged liver. The spleen demonstrates multiple small hypoattenuating lesions (arrows).

**Figure 3:**
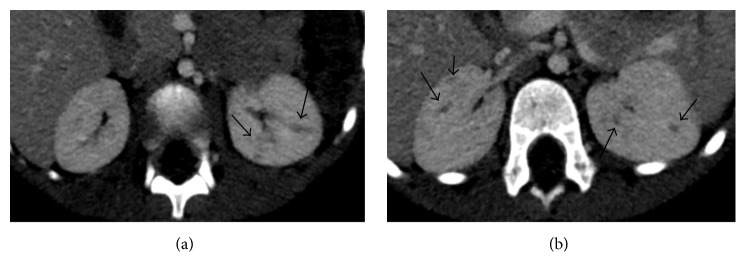
Two axial contrast-enhanced CT images through the level of the kidneys demonstrate small bilateral hypoattenuating renal lesions.

On hospital day 2, a complete abdominal ultrasound demonstrated an echogenic liver which was slightly enlarged to 11 cm in the craniocaudal dimension at the right midclavicular line. The spleen was also enlarged to 7.8 cm and contained several small hypoechoic foci (Figures 1(a) and 1(b)). A contrast-enhanced CT was then recommended for further evaluation which showed small, poorly defined hypoattenuating lesions that were seen throughout the spleen suspicious for microabscesses (Figures 2(a) and 2(b)). Additionally, small, poorly defined hypoattenuating lesions were seen within the bilateral renal cortices, findings which were also compatible with microabscesses (Figures 3(a) and 3(b)).

Epstein-Barr virus, cytomegalovirus, toxoplasmosis, HIV, histoplasmosis titers, and purified protein derivative tests were all negative. On hospital day 3, the* Bartonella henselae* IgG was positive at >1 : 1024, consistent with presence of IgG antibody to* Bartonella henselae*, suggestive of current or prior infection.* Bartonella henselae* IgM was also positive at 1 : 128, suggestive of current or recent infection. Given the CT and ultrasound findings, the constellation of findings was consistent with an active* Bartonella henselae* infection with involvement of the spleen, kidneys, and liver.

Following these results, the patient was treated with oral rifampin 150 mg by mouth twice daily for fourteen days and trimethoprim-sulfamethoxazole 150 mg by mouth twice daily for fourteen days, which was well tolerated. The patient's fever and other symptoms resolved during her hospital stay and she was discharged on continuation of the oral antibiotic regimen. At the time of discharge on hospital day 5, the patient had defervesced and her inflammatory markers had decreased, with a C-reactive protein of 2.61 at the time of discharge. Her subsequent outpatient follow-up in the infectious disease clinic 11 days after discharge demonstrated complete resolution of her symptoms.

## 3. Discussion

Though well-known as a cause of infection,* Bartonella henselae* is infrequently discussed in the radiology literature. In this report, we present a case of a pediatric patient with* Bartonella henselae* infection resulting in splenomegaly and microabscesses of the spleen and kidneys as noted on CT and ultrasound; to the best of our knowledge, this is the first report of this constellation of findings on radiologic studies in a pediatric patient.

It was only as recently as 1992 that the pathogen* Bartonella henselae* was isolated [5].* Bartonella henselae* is an intracellular bacillus which can be identified on Warthin-Starry silver stains [1]. The primary reservoir for* Bartonella henselae* is cats, with half of pet cats demonstrating antibodies against the bacteria [1]. Stray cats have an even higher incidence of bacteremia and seropositivity [6]. The infection spreads via an arthropod vector,* Ctenocephalides felis* or the “common” cat flea [1, 5]. The bacterium may be able to survive in the feces of the cat flea for up to a week [5]. Cats associated with cat-scratch disease are highly likely to be bacteremic with* Bartonella henselae*; however, even 28% of cats without known associations to cat-scratch disease or bacillary angiomatosis have* Bartonella henselae* bacteremia [7]. Prevention of infection includes hand-washing after handling pets, with particularly close attention to any bites or scratches one may endure; however, the most effective prevention is the eradication of fleas [8].

Upon infection, the bacteria invade many types of cells; most commonly this involves dendritic cells but also includes CD34+ progenitor cells, erythrocytes, pericytes, and perhaps most importantly endothelial cells [1, 5].* Bartonella henselae* invasion of endothelial cells in both reservoir and incidental hosts [9] results in vascular proliferation [10]. This is accomplished both by inhibiting endothelial cell apoptosis [10] and by stimulating the host to produce vascular endothelial growth factor (VEGF) which results in endothelial cell stimulation; subsequently, these endothelial cells promote proliferation of* Bartonella henselae* [11].

The clinical presentation of* Bartonella henselae* infection is dependent on the immune status of the affected host. Immunocompetent patients most commonly present with fever which may be protracted, localized lymphadenopathy and hepatosplenic disease. Typical cat-scratch disease includes fever and localized lymphadenopathy usually only affecting a single lymph node [1]. The patient may also note a nontender papule which develops a few days following the inciting scratch [6]. Although it is often thought of as only a self-limiting disease,* Bartonella henselae* can also result in chronic bacteremia, even in immunocompetent patients, who may be entirely asymptomatic or may present with mild or fluctuating symptoms, including fatigue, myalgias, headaches, generalized pains, and insomnia [5, 12].* Bartonella henselae* infection is also one of the most common causes of a fever of unknown origin in a pediatric patient, with some studies showing it to be the third most common cause of a prolonged fever in children [13].

Immunodeficient patients may manifest infection as cutaneous bacillary angiomatosis [14], with cutaneous or subcutaneous vascular tumors which may be painful. They may be solitary or few or exist in hundreds [6]. If not adequately treated, these lesions may disseminate throughout the entire body to involve the respiratory tract and gastrointestinal tract, involve the heart to cause endocarditis, and infect the CNS resulting in meningitis and brain abscesses [14]. Bacillary peliosis is another manifestation of* Bartonella henselae* infection in immunocompromised patients, typically those with AIDS [6]. It presents with cystic blood-filled spaces in the spleen and/or liver [6].

Diagnosing* Bartonella henselae* infection can be quite challenging, in part because many of the signs and symptoms are nonspecific and can be seen in other infections including cytomegalovirus infection, HIV infection, toxoplasmosis, and Epstein-Barr virus infection, as well as noninfectious etiologies including malignancies such as lymphoma [15]. The path to diagnosis begins with physical examination and history including presence or absence of lymphadenopathy and any prior exposure to cats [15]. The best test to be performed initially is serology either by indirect fluorescent assay or by enzyme-linked immunosorbent assay [15]. Unfortunately, reports on the efficacy of these are variable, with some showing serology to have a very low sensitivity and others reporting a high sensitivity [15–17]. The sensitivity of serologic tests also varies by which immunoglobulin is evaluated, with the sensitivity of IgM much lower than that of IgG [18]. Conversely, specificity is much higher in IgM tests than IgG [18]. With this in mind, it is important to remember that many patients do not mount a significant antibody response to* Bartonella henselae* infection [16]. An additional possible confounding factor with serology is that results may be positive due to a remote infection. However, immunoglobulin G levels >1 : 256 are strongly suggestive of either an acute or recent* Bartonella henselae* infection. In the setting of equivocal serology but a persistent clinical suspicion for* Bartonella henselae* infection, lymph node biopsy can be performed with polymerase chain reaction to identify DNA fragments [15, 16]. The use of enriched cultures and PCR from a number of sources including blood and CSF can enhance both sensitivity and specificity of diagnosing* Bartonella henselae* infection [5].

On radiologic imaging, hepatosplenic involvement of* Bartonella henselae* is becoming more widely discovered due to increased use and accuracy of radiological studies. These lesions are seen as hypoattenuating on CT and hypoechoic on ultrasound, which correspond to necrotizing granulomata on pathology [1]. These tend to range in size from being as small as 3 mm to being as large as 3 cm [3, 6]. On contrast-enhanced CT, these lesions may remain hypoattenuating or may show mild enhancement [6]. Splenomegaly in the absence of granulomatous involvement may be seen in 12% of patients [3]. In a study of 11 pediatric age patients with hepatosplenic* Bartonella henselae* infection, only two had hepatomegaly and two had splenomegaly; all patients in this series had splenic microabscesses and the majority had hepatic microabscesses [2]. Only 64% of these patients presented with complaints of abdominal pain [2].

The first reported case of bilateral renal microabscesses secondary to* Bartonella henselae* infection was reported in 2010 [4], which presented with a normal urinalysis and renal function panel. Renal microabscesses can be shown by contrast-enhanced CT scan, even in the setting of normal kidney function [4]. However, the most common manifestation of renal involvement of* Bartonella henselae* is glomerulonephritis [1].

In cats, no antibiotic has proven routinely effective for the treatment of* Bartonella henselae*, with incomplete treatment responses to multiple antibiotic agents. Rather, in cats the optimal management is the eradication of fleas [19]. A monthly topical application of 10% imidacloprid-1% moxidectin has been shown to decrease flea transmission [20]. Other drugs including Selamectin have shown to reduce the number of cat fleas [21]. In human infection, the clinical manifestations of* Bartonella henselae* dictate the appropriate therapeutic management; for instance, typical cat-scratch disease is often self-limited and poorly responsive to antimicrobial treatment [1], whereas hepatosplenic disease has been shown to be successfully treated with trimethoprim-sulfamethoxazole, rifampin, ciprofloxacin, and gentamicin. However, it is unknown which antibiotic is optimal for treatment due to a lack of controlled trials [1]. Treatment of* Bartonella henselae* infection is also dependent on the patient's immune status, with immunocompetent patients requiring only 2–4 weeks of therapy. In immunocompromised patients, even prolonged antibiotic courses of 6 weeks may subsequently relapse and demonstrate bacteremia [19]. As a result, these patients may require lifelong antimicrobial therapy [14].
